# Microbial Dynamics in Wine Production

**DOI:** 10.3390/microorganisms9040700

**Published:** 2021-03-28

**Authors:** Rosanna Tofalo

**Affiliations:** Faculty of Bioscience and Technology for Food, Agriculture and Environment, University of Teramo, via R. Balzarini 1, 64100 Teramo, Italy; rtofalo@unite.it

The Special Issue “Microbial dynamics in wine production” collects nine original research articles and a review concerning wine production, a complex process where microbes have a decisive role. Each wine has distinct characteristics deriving from the environment, weather and climate where the vines grow, vineyard management practices and the microbes, whose presence can modulate the composition and quality of wine.

Fermentative yeasts, such as *Saccharomyces cerevisiae*, non-*Saccharomyces* yeasts and lactic acid bacteria (LAB, predominantly *Oenococcus oeni*) release different metabolites during fermentation which have an impact on wine volatile profile. Wine regionality is partially influenced by microbial communities distributed in various viti-vinicultural areas where grape microorganisms can be transferred to the winery affecting wine chemical composition and influencing its quality, even at the regional scale [1].

Microbial dynamics during wine fermentation have been deeply explored by advanced metabolic and genetic-based methodologies, highlighting the impact on wine quality and regional variation.

Kioroglou et al. [2] used barcode sequencing and an innovative compositional data analysis to underline the importance of the vineyard microbiome composition over the regional wine typicity. They studied eukaryotic population in the grape berries at different ripening states in four Australian vineyards at two different geographical and environmental conditions. Their findings underlined that fungal composition and diversity were mainly influenced by the vineyard region while the grape variety or the ripening state had less impact. Fungal community composition varied significantly across the different vineyards.

Vineyard and soil affect grape microbiota composition, which slightly changes when berries are crushed to obtain the must. Non-*Saccharomyces* yeasts are mainly present in the first stages of fermentation, and along with *S. cerevisiae* yeasts, can participate in multi-species fermentations.

The employment of multi-species starter cultures has gained attention in modern winemaking for the capacity to improve complexity and wine attributes. Selection of the most performant species/strains is crucial to assure wine quality and safety.

In the paper of Tufariello et al. [3], the effect of co-inoculation of *Starmerella bacillaris* (syn. *Candida zemplinina*), *S. cerevisiae* and *Lactoplantibacillus plantarum* for the industrial production of Negramaro wine was evaluated. This autochthonous multistarter culture has been proved to be a valid solution to enhance the peculiar features of this wine, mimicking the tendency of a return to spontaneous fermentation.

The use of non-*Saccharomyces* yeasts in wine fermentation required a deep knowledge of their metabolism and nutrient requirements. Carbon and nitrogen compounds are involved in the metabolism and growth of yeasts influencing the fermentation outcome and the production of volatile compounds.

Roca-Mesa et al. [4] evaluated the nitrogen preferences of some non-*Saccharomyces* wine yeasts (*Torulaspora delbrueckii*, *Lachancea thermotolerans*, *Starm. bacillaris*, *Hanseniaspora uvarum*, and *Metschnikowia pulcherrima*). This study highlighted a species-dependent attitude in nitrogen consumption and preferences. It is crucial to control the nitrogen availability during fermentations since it can be consumed by non-*Saccharomyces* before *S. cerevisiae* inoculation causing stuck or sluggish fermentations.

Other than nutrition, the characteristics of the strain inoculation method can influence the fermentation outcome. With regard to malolactic fermentation (MLF), these parameters are even more important because of its unpredictable nature. Lombardi et al. [5] evaluated different inoculation approaches and tested the performance of a *Lpb. plantarum* strain to conduct MLF. This study highlighted that correct management of the inoculation strategies can determine both the success of the MLF and the improvement of the sensorial characteristics of the wines.

Microbial dynamics and metabolic activities are crucial for fermented beverages such as sparkling wines and beer. Hu et al. [6] demonstrated how yeast proteinase A (Pep4p) influences glycolytic metabolism and stress responses in *S. cerevisiae* and can hydrolyze lipid transport protein (Ltp1), which is the major protein for stabilizing foam in sparkling wine and beer. Proteomic and molecular studies identified 35 proteins which were downregulated under the condition of Pep4p deficiency. This study highlighted that Pep4p could regulate *S. cerevisiae* physiology and metabolism under nitrogen starvation.

During alcoholic fermentation, yeasts face a variety of stressing conditions influencing their metabolic performances and proliferation rates. Sommer [7] underlines how the use of flow cytometry could be applied to fermentation monitoring to visualize how and when wine yeast responds to stress factors caused by the changing conditions of alcoholic fermentation. In this research, correlations between intracellular macromolecules such as glycogen and neutral lipids and yeast viability as well as stress responses were shown.

Non-*Saccharomyces* wine yeasts can positively influence wine traits but, some species, are known to spoil wine producing films, high volatile acidity or undesirable volatile compounds. Wine spoilage yeasts mainly belong to the following genera *Dekkera/Brettanomyces*, *Candida*, *Hanseniaspora*, *Pichia*, *Metschnikowia*, *Saccharomycodes*, *Schizosaccharomyces* and *Zygosaccharomyces.* They can originate from soil, water, insects, vines, or grapes. Some species have the ability to adhere and survive on abiotic surfaces, such as cork stoppers or the bottling plants. Rodríguez-Andrade et al. [8] isolated 27 different fungi from deteriorated sparkling wine and cork stoppers and found a new genus (*Dactylodendron*) and eight new species (*Cladophialophora recurvata, Dactylodendron ebriosum, Dactylodendron pluriseptatum, Kirschteiniothelia ebriosa, Kirschteiniothelia vinigena, Rasamsonia frigotolerans, Talaromyces speluncarum* and *Talaromyces subericola*). The fungi found in sparkling wines were also present on the cork stoppers and/or are part of the environment of the cellar.

Perpetuini et al. [9] studied some yeasts isolated from filter membranes used for wine quality control. Strains were tested for their ability to adhere to abiotic surfaces, and for their resistance to potassium metabisulfite and some cleaning agents generally used in cellars to reduce/prevent wine spoilage. This study revealed novel information on the diversity and resistance of potential contaminant yeasts encountered in the wine environment.

Yeasts can develop as biofilms, which are more difficult to eradicate than planktonic cells. Biofilm formation helps yeasts to endure in the environment permitting access to oxygen and, growth on non-fermentable carbon sources, such as equipment surfaces. Moreover, biofilms can be exploited in food biotechnology when microbial interactions are investigated. Ly et al. [10] studied engineered synthetic microbial communities through a selective biofilm cultivation device to produce fermented beverages. This biofilm cultivation device could be used to promote structuration of microbial species within communities (fungi, yeast, and bacteria), modulating the ratio among ethanol, organic acid and volatile compounds production.

Microorganisms can be a useful resource to improve the sustainability aspects of winemaking, and the production chain. The review by Nardi [11] underlined the potential of microorganisms to be exploited as sustainable tools. The author comprehended several positive effects, including energy savings, reduction in chemical additives such as sulfites, and waste treatment.

This Special Issue is interesting for both the scientific community and winemakers. In fact, in recent years, the oenological industry is facing new challenges, becoming a sector with constant changes and innovations. Studies on genetic and metabolic traits of wine microorganisms as well as oenological practices are essential to produce new and differentiated wine styles which reflect the characteristics of a specific viti-vinicultural area.
